# Gold Nanoparticles sensitize pancreatic cancer cells to gemcitabine

**DOI:** 10.15698/cst2019.08.195

**Published:** 2019-07-31

**Authors:** Yanyan Huai, Yushan Zhang, Xunhao Xiong, Shamik Das, Resham Bhattacharya, Priyabrata Mukherjee

**Affiliations:** 1 Department of Pathology, the University of Oklahoma Health Sciences Center, Oklahoma City, Oklahoma, USA.; 2 Peggy and Charles Stephenson Cancer Center, the University of Oklahoma Health Sciences Center, Oklahoma City, Oklahoma, USA.

**Keywords:** gold nanoparticle, gemcitabine, drug resistance, cancer stem cell, EMT

## Abstract

Pancreatic ductal adenocarcinoma (PDAC) is one of the deadliest solid cancers with dismal prognosis. Several mechanisms that are mainly responsible for aggressiveness and therapy resistance of PDAC cells include epithelial to mesenchymal transition (EMT), stemness and Mitogen Activated Protein Kinase (MAPK) signaling. Strategies that inhibit these mechanisms are critically important to improve therapeutic outcome in PDAC. In the current study, we wanted to investigate whether gold nanoparticles (AuNPs) could sensitize pancreatic cancer cells to the chemotherapeutic agent gemcitabine. We demonstrated that treatment with AuNPs of 20 nm diameter inhibited migration and colony forming ability of pancreatic cancer cells. Pre-treatment with AuNPs sensitized pancreatic cancer cells to gemcitabine in both viability and colony forming assays. Mechanistically, pre-treatment of pancreatic cancer cells with AuNPs decreased gemcitabine induced EMT, stemness and MAPK activation. Taken together, these findings suggest that AuNPs could be considered as a potential agent to sensitize pancreatic cancer cells to gemcitabine.

## INTRODUCTION

Pancreatic ductal adenocarcinoma (PDAC) is a highly lethal disease. The mortality rate closely parallels that of incidence [1, 2]. The current standard of care for advanced stage PDAC patients includes combination treatment with Nab-paclitaxel and gemcitabine. The currently reported five-year survival rate for pancreatic cancer is 9% [3]. New therapeutic strategies are urgently required to improve poor prognosis in PDAC patients.

Epithelial-to-mesenchymal transition (EMT) [4, 5], stemness [6, 7] and Mitogen-activated protein kinases (MAPK) activation [8] are among the key mechanisms responsible for poor outcome in PDAC. EMT is deemed as a key driver in the progression and metastasis of cancer [9, 10]. Gemcitabine treatment upregulated expression of EMT markers in pancreatic cancer cells *in vitro* [11] and is associated with resistance to anti-EGFR therapy [12], indicating a role of EMT in chemotherapy resistance. EMT had been reported to induce stemness in pancreatic cancer cells causing drug resistance [13] whereas its inhibition alleviated drug resistance [14, 15].

Growth factors (GFs) mediated activation of MAPK functions as a key signaling hub promoting tumor growth, metastasis and therapy resistance. Previously, we demonstrated that gold nanoparticles (AuNPs) disrupted GF-mediated signaling and reversed EMT leading to inhibition of tumor growth in pancreatic and ovarian cancer [16, 17]. Accumulating evidence suggests that AuNPs may function as a new therapeutic agent. AuNPs treatment altered the secretome of pancreatic cancer cells due to the deprivation of key hub proteins which can regulate other secretory proteins and additionally through RIDD-dependent cleavage of ER-localized mRNA induced by ER stress [16]. Kim *et al.* reported that 20 nm AuNPs passed through the blood-retinal barrier (BRB) and distributed in neurons, endothelial cells and peri-endothelial glial cells without causing any cell structural abnormality or cytotoxicity [18]. Furthermore, pretreatment with AuNPs could enhance the radiation effect in ocular melanoma [19]. In an established collagen-induced arthritis (CIA) model in rats, treatment with the pure 13 nm and 50 nm AuNPs decreased joint swelling by 49.7% (P<0.002) and 45.03% (P<0.01), respectively [20]. In our previous study, AuNPs pre-treatment sensitized ovarian cancer cells to cisplatin [21]. In the present study, we wanted to exploit the unique self-therapeutic property of AuNPs and investigate if AuNPs could sensitize PDAC cells to gemcitabine. We also delved into the mechanistic aspect of this phenomenon by investigating the effect of AuNPs treatment on EMT signaling, stemness and MAPK signaling.

## RESULTS

### AuNPs decrease the 2D colony, 3D sphere formation and migration ability of pancreatic cancer cells

**Figure 1:**
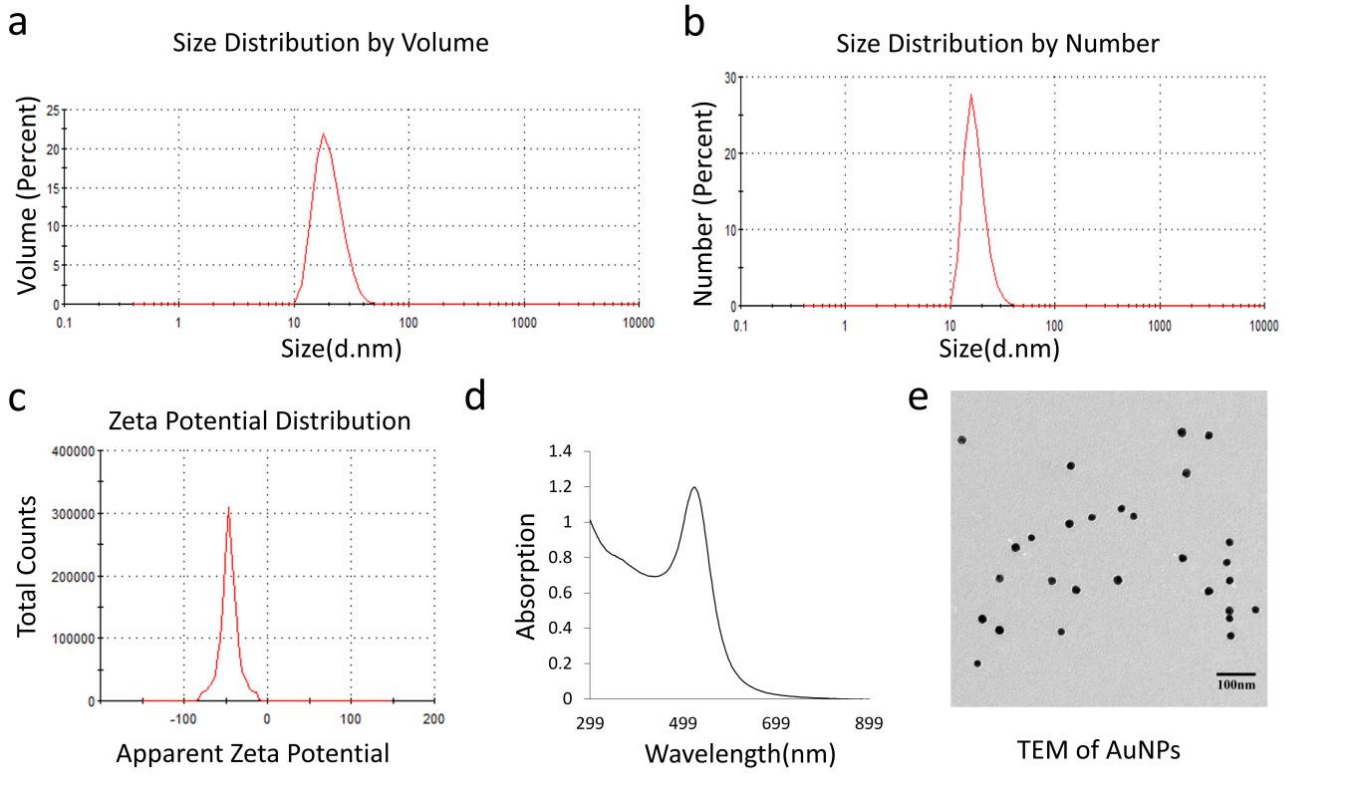
FIGURE 1: Characterization of 20 nm AuNPs. The average size of the prepared AuNPs was measured with DLS and TEM. The distribution peaks were at 20.23 nm and 18.01 nm according to the volume and number distribution respectively **(a and b)**, and the zeta potential of AuNPs was about -45.9±11.2 mV **(c)** (Malvern DTS1061). The absorption peak was at about 522 nm **(d)**. The TEM image showed the size and shape of AuNPs **(e)**.

**Figure 2:**
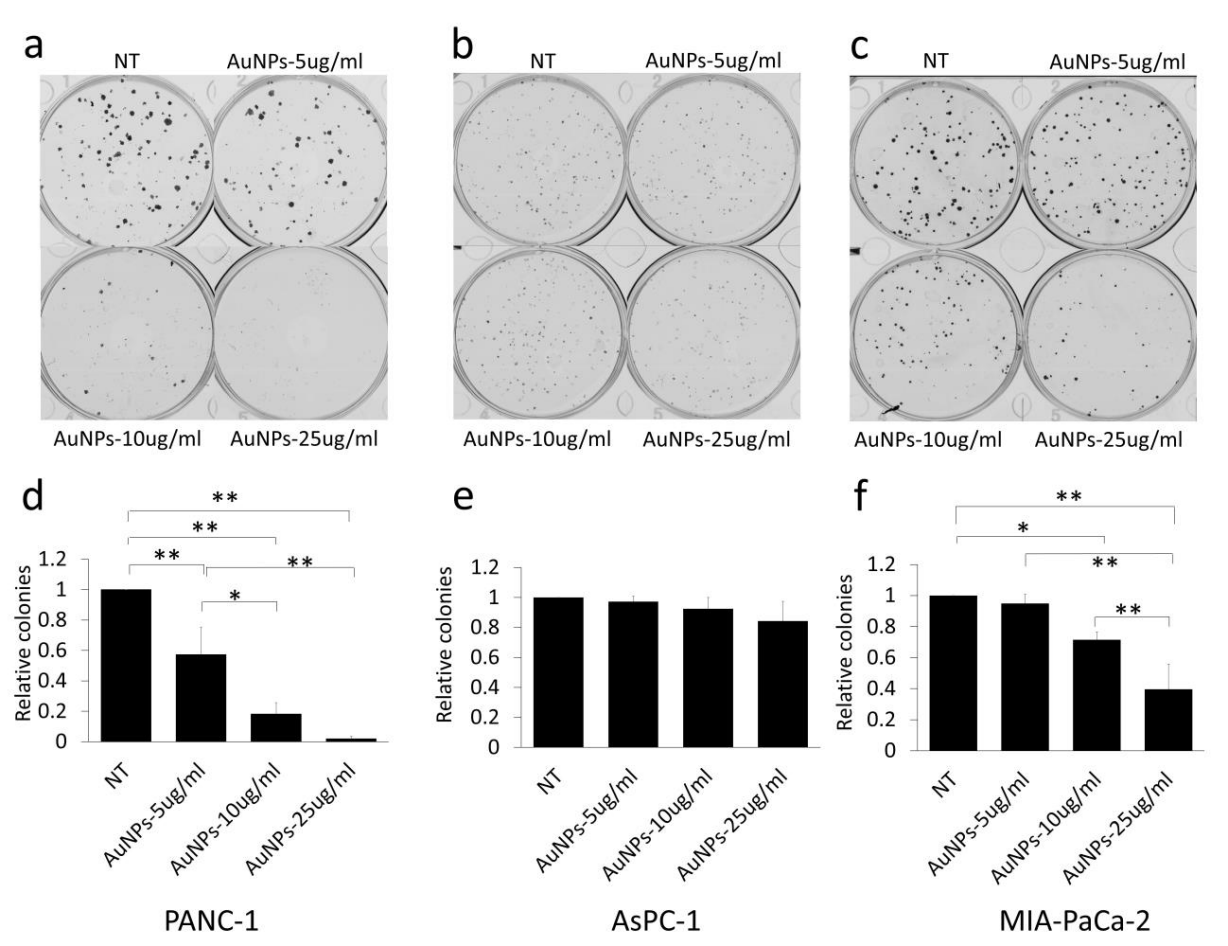
FIGURE 2: Dose dependent inhibition of colony formation in AuNPs treated pancreatic cancer cell lines. Three pancreatic cancer cell lines, PANC-1 **(a)**, AsPC-1 **(b)** and MIA-PaCa-2 **(c)**, were first seeded with the complete medium (200 cells/well) and 24 h after seeding, treated with different concentrations of AuNPs (5 μg/ml, 10 μg/ml or 25 μg/ml). 7-10 days later, the colonies were stained with crystal violet and the numbers of colonies were counted (Gelcounter). **(a-c)** The colonies formed with different pancreatic cancer cells with different concentration of AuNPs treated. **(d-f)** Relative colony numbers corresponding to a-c. Independent experiments were repeated for at least three times and each time, at least triplicate wells were used. Values are means ± SD and statistical analysis were done using one-way ANOVA. *P≤0.05, **P≤0.01.

**Figure 3:**
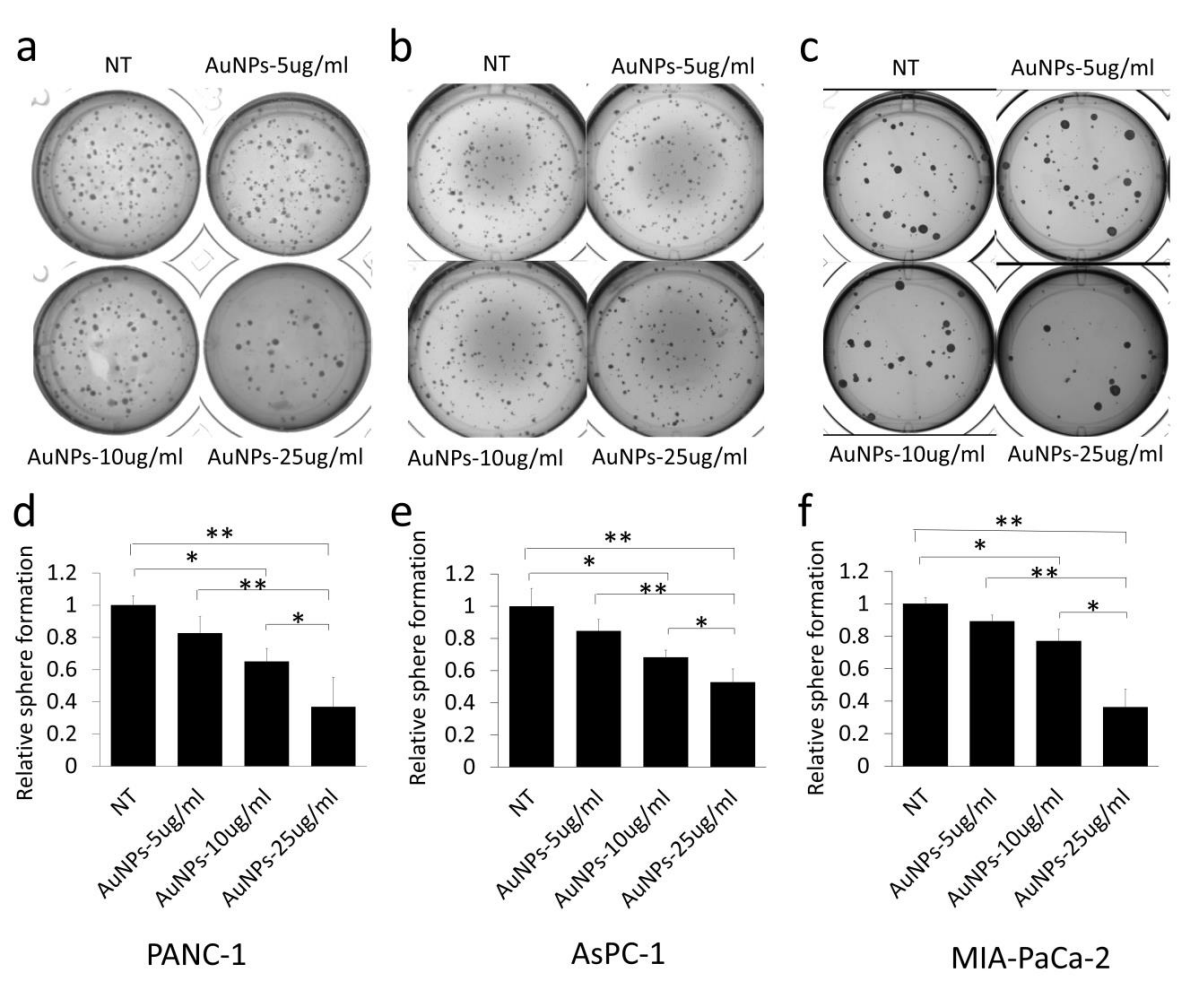
FIGURE 3: Dose dependent inhibition of 3D sphere formation in AuNPs treated pancreatic cancer cell lines. PANC-1 **(a)** (600 cells/well), AsPC-1 **(b)** (600 cells/well) and MIA-PaCa-2 **(c)** (400 cells/well) were suspended in complete medium and then mixed with equal volumes of matrigel, the cell mixture was added on the top of the lower layer of solid matrix (complete medium: matrigel=1:1) in 24 wells plate. On the top the mixture, 1 ml of complete medium with different concentrations of AuNPs (5 μg/ml, 10 μg/ml or 25 μg/ml) was added. The sphere numbers were counted 20-40 days after seeding. **(a-c)** The spheres formed with different pancreatic cancer cells with different concentration of AuNPs treated. **(d-f)** Relative sphere numbers corresponding to a-c. Independent experiments were repeated for at least three times and each time, at least triplicate wells were used. Values are means ±SD and statistical analysis were performed using one-way ANOVA. *P≤0.05, **P≤0.01.

20 nm AuNPs were synthesized by the citrate reduction method [16]. We physicochemically characterized AuNPs by dynamic light scattering (DLS), Zeta potential, transmission electron microscopy (TEM) and UV-visible spectroscopy. DLS measurements demonstrated that AuNPs with hydrodynamic diameter (HD) of ~ 20.23 nm ± 5 nm (based on volume) **(Fig. 1a)** were formed by this method having a net negative charge of -45.9 ± 11.2 mV **(Fig. 1c)** as determined by the Zeta potential measurements. UV-Visible spectra of AuNPs exhibited an absorption maxima at ~522 nm **(Fig. 1d)**, indicative of spherical AuNPs formed by this method. TEM further confirmed that AuNPs of ~ 20 nm in diameter were formed by this method **(Fig. 1e)**. Previously, we demonstrated that 20 nm AuNPs inhibited the proliferation of the pancreatic cancer cells *in vitro* [16]. Since colony forming ability of cancer cells is considered as a measure of stem-like properties of cancer cells that are partially responsible for drug resistance and poor outcome, we wanted to investigate if AuNPs could inhibit clonal growth of pancreatic cancer cells. We performed both two dimensional (2D) and three dimensional (3D) sphere formation assays. For the 2D colony formation assay, cells were first seeded and 24 h later treated with different doses of AuNPs 5 μg/ml, 10 μg/ml or 25 μg/ml and final measurements were done 7-10 days after AuNPs treatment. It is evident from **Figure 2** that AuNPs decreased the colony forming ability of pancreatic cancer cells in a dose dependent manner compared to non-treated controls. Treatment with AuNPs of 5 μg/ml, 10 μg/ml or 25 μg/ml decreased colony numbers in PANC-1 cells by 43%, 82% and 99% **(Fig. 2a** and **2d)**, respectively, and in MIA PaCa-2 cells by 5%, 28% and 60%, respectively. Interestingly, 2D colony formation ability of AsPC-1 cells was not altered by AuNPs treatment **(Fig. 2b** and **2e)**. Treatment with AuNPs of 5 μg/ml, 10 μg/ml or 25 μg/ml decreased 3D colony formation by ~ 25, 50 or 70% respectively in PANC-1 cells **(Fig. 3a** and **3d)**, 20, 40 and 50% in AsPC1 cells **(Fig. 3b** and **3e)**, respectively and 15, 30 and 70%, respectively, in MiaPaca2 cells **(Fig. 3c** and **3f)**. Thus, AuNPs inhibited colony forming ability of pancreatic cancer cells, suggesting potential of AuNPs to sensitize pancreatic cancer cells to chemotherapeutics.

**Figure 4:**
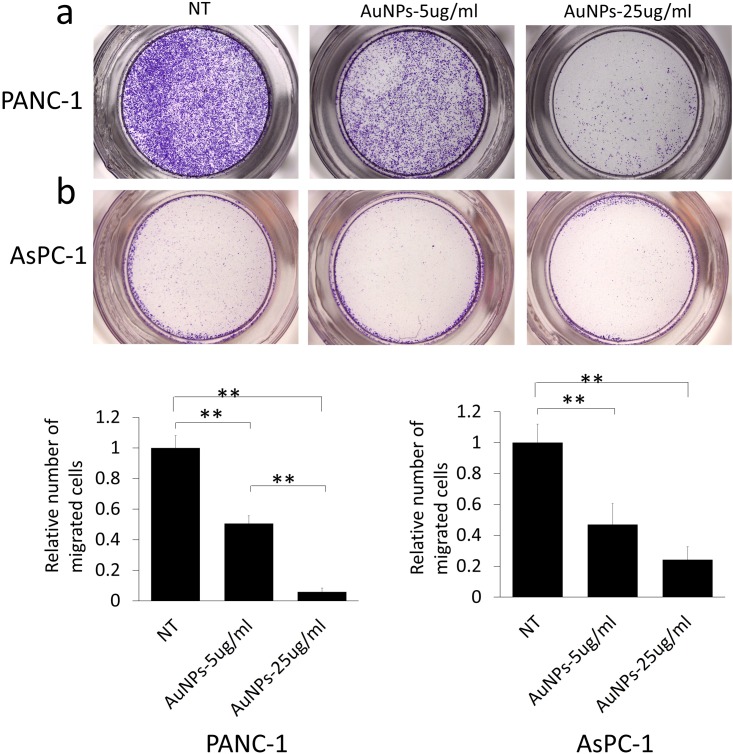
FIGURE 4: Dose dependent inhibition of the migration in AuNPs treated pancreatic cancer cell lines. PANC-1 **(a)** and AsPC-1 **(b)** were first starved for 24 h after seeding, and then treated with AuNPs (5 μg/ml or 25 μg/ml) for 48 h under the starvation condition. After the treatment, cells were collected and seeded in the upper chamber of the transwell separately at the concentration of 1×10^5^/well; cell culture medium including 1% FBS was added into the lower chamber. 16 h later, the non-migrated cells were removed with a cotton swab, and the migrated cells were stained and quantified **(c, d)**. Independent triplicate experiments were repeated. Values are means ± SD and statistical analysis were performed using one-way ANOVA. *P≤0.05, **P≤0.01.

Since migration plays a crucial role in the metastasis and therapy resistance, next we wanted to investigate whether AuNPs could be used to inhibit migration of pancreatic cancer cells. The cells were first primed by serum starvation followed by AuNPs treatment for 48 h. Non-treated but serum starved cells were used as controls (NT). After the treatment, cells were seeded in the transwell inserts to test the migration ability. Compared with the NT group, treatment with AuNPs (5 μg/ml or 25 μg/ml) reduced migration by 50% or 95% in PANC-1 **(Fig. 4a)**, respectively, while migration of AsPC-1 cells was reduced by 53% or 76% **(Fig. 4b)**, respectively. Thus, AuNPs inhibited migration of pancreatic cancer cells in a dose-dependent manner.

### AuNPs sensitize pancreatic cancer cells to gemcitabine

**Figure 5:**
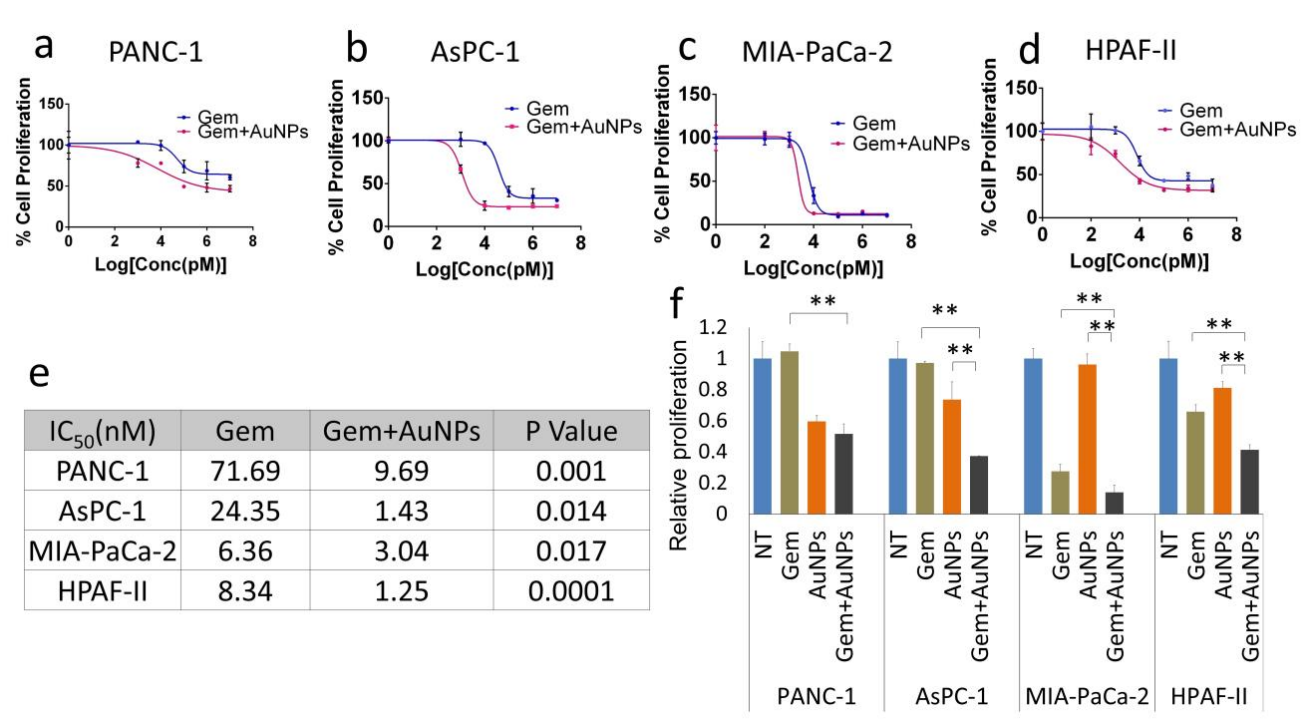
FIGURE 5: AuNPs decrease IC_50_ of gemcitabine on pancreatic cancer cell lines. After seeding, cells were first treated with/without AuNPs (25 μg/ml) for 24 h in the complete medium, followed by gemcitabine for 72 h. The proliferation of PANC-1 **(a)**, AsPC-1 **(b)**, MIA-PaCa-2 **(c)** and HPAF-II **(d)** were measured with MTT and the IC_50_ were calculated with Prism. The IC_50_ value are shown in the table **(e)**. The proliferation of each cell line at the concentration of 10 nM of gemcitabine was calculated **(f)**. Independent triplicate experiments were repeated and each time, at least triplicate wells were used. Values are means ±SD and statistical analysis were performed using one-way ANOVA.

**Figure 6:**
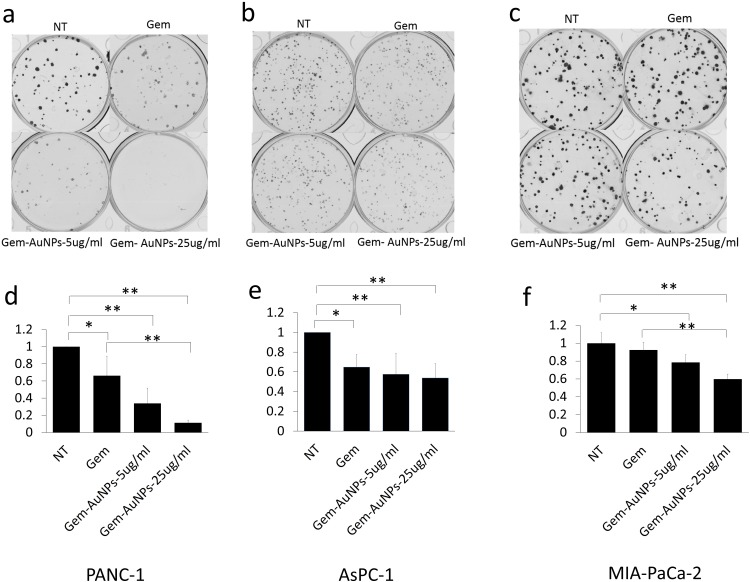
FIGURE 6: AuNPs sensitize pancreatic cancer cells to gemcitabine. PANC-1 **(a)**, AsPC-1 **(b)** and MIA-PaCa-2 **(c)** were first seeded (200 cells/well) in complete medium in 6 wells plates. 24 h later, cells were treated with gemcitabine (5 nM for Panc1, 2.5 nM for Aspc1 and 0.7 nM for Miapaca2) with/without AuNPs at different concentrations (5 μg/ml or 25 μg/ml). 7-10 days later, the colonies were stained with crystal violet and the numbers of colonies were counted **(d-f)**. Independent experiments were repeated for at least three times and each time, at least triplicate wells were used. Values are means ± SD and statistical analysis were performed using one-way ANOVA. *P≤0.05, **P≤0.01.

Ability to form clonal growth is an indication of cancer cell stemness and drug resistance. Since AuNPs inhibited clonal growth of pancreatic cancer cells, we investigated whether AuNPs could sensitize pancreatic cancer cells to gemcitabine. Half-maximal inhibitory concentration (IC_50_) was used as informative measurement of the efficacy of a drug. We next utilized four pancreatic cancer cell lines: PANC-1, AsPC-1, MIA PaCa-2 and HPAF II to investigate whether AuNPs decrease the IC_50_ of gemcitabine treatment. Cells were treated with/without AuNPs for 24 h, and then with gemcitabine for 72 h. The proliferation of each group was measured with MTT and the IC_50_ was calculated. As shown in **Figure 5**, compared with the gemcitabine only treatment group, pretreatment with AuNPs reduced IC_50_ of gemcitabine 7.4 times, 17 times, 2.1 times and 6.7 times in PANC-1, AsPC-1, MIA PaCa-2 and HPAF II cell lines, respectively **(Fig. 5a-e)**. Moreover, the relative proliferation in combination groups was lower than that of the AuNPs groups in PANC-1 and AsPC-1 cells even when gemcitabine alone had no significant effect at the concentration of 10 nM **(Fig. 5f)**. These results suggest that AuNPs show a significant synergistic effect with gemcitabine (10 nM) to inhibit the proliferation of four pancreatic cancer cell lines **(Fig. 5f)**. Taken together, these results demonstrate AuNPs could sensitize pancreatic cancer cells to gemcitabine. To further validate this observation, we performed a 2D colony formation assay with the treatment of AuNPs (5 μg/ml or 25 μg/ml) and/or gemcitabine. While treatment with AuNP of 5μg/ml had a marginal effect on the 2D colony forming ability, AuNPs-gemcitabine combined treatment significantly decreased the colony numbers compared to the gemcitabine only treatment in PANC-1 from 66% to 34% and MIA PaCa-2 from 92% to 78% cell lines at the concentration of 5 μg/ml **(Fig. 6a-f)**. Thus, AuNPs sensitized pancreatic cancer cells to gemcitabine.

### AuNPs prevent gemcitabine-induced EMT

**Figure 7:**
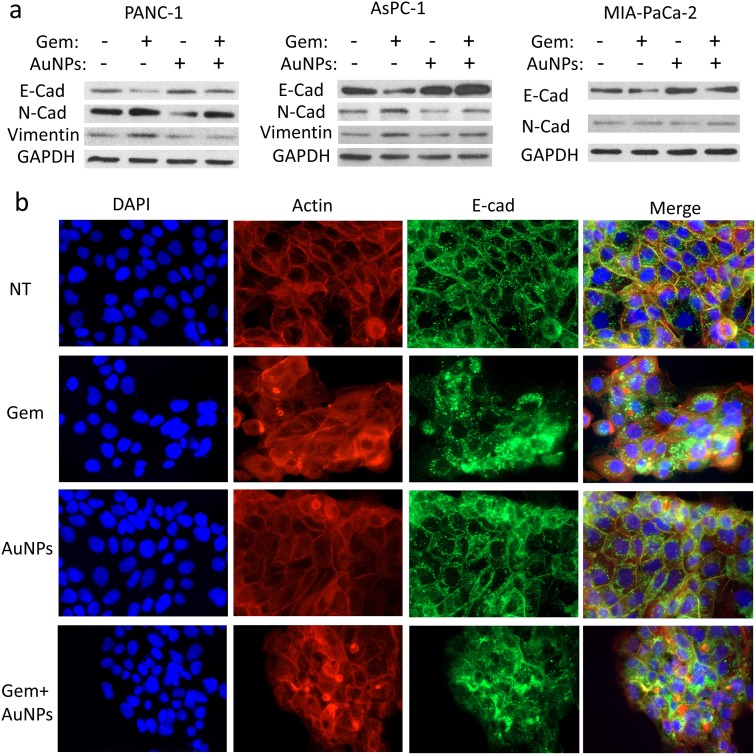
FIGURE 7: AuNPs reverse EMT induced by gemcitabine on pancreatic cancer cells. Pancreatic cancer cell lines PANC-1, AsPC-1 and HPAF-II were first serum-starved for 24 h and were then divided into four groups for the treatment under the serum-starved condition: 1) NT group, 2) treated with gemcitabine (100 nM) for 72 h, 3) treated with AuNPs (25 μg/ml) for 24 h, 4) first treated with AuNPs (24 h) and then with gemcitabine (100 nM, 72h). **(a)** Western blot analyses of EMT markers following the afore mentioned treatment. GAPDH (1:10000) was used as the loading control. E-cad, E-cadherin (1:1000); N-cad, N-Cadherin (1:1000); Vim, Vimentin (1:1000). **(b)** AuNPs reverses the gemcitabine-induced relocalization of E-Cadherin in HPAF-II. At the end of the treatment, cells were fixed, stained with anti-E-cadherin antibody (1:200) followed by Alexa Fluor 488-conjugated secondary antibody (1:500), then stained with the Alexa Fluor 568-Phalloidin (1:1000) and DAPI. The localization of E-Cadherin, F-actin and nuclei were visualized by immunofluorescence microscopy. Independent experiments were repeated for at least three times.

EMT is an important mechanism underlying the initiation of tumor invasion and metastasis [22, 23], Furthermore EMT is a possible cause for drug resistance in cancer cells [7]. Our previous work showed that AuNPs could reverse EMT and sensitize ovarian cancer cells to cisplatin [17, 21]. Here, we hypothesized that EMT might be one of the possible mechanisms by which pancreatic cancer cells become resistant to gemcitabine. We treated three pancreatic cancer cells PANC-1, AsPC-1 and HPAF II either with gemcitabine (72 h), AuNPs (96 h), or first with AuNPs (24 h) and then with gemcitabine (72 h). The expression of several EMT markers were then determined by western blotting. Treatment with gemcitabine only significantly up-regulated the expression of mesenchymal markers like N-cadherin and Vimentin, while down-regulated the epithelial marker E-cadherin **(Fig. 7a)**. In line with our previous findings [17], treatment with AuNPs reduced mesenchymal transition by down-regulating the expression of mesenchymal markers (N-cadherin and Vimentin) and up-regulating the epithelial marker (E-cadherin). Interestingly, pre-treatment with AuNPs significantly blunted the EMT effect induced by gemcitabine **(Fig. 7a)**. To further clarify the role of AuNPs in reversing the EMT effect, we performed fluorescence studies with the pancreatic cancer cell line HPAF-II to observe the morphological transition. E-cadherin and F-actin were stained with the corresponding antibodies after the treatment **(Fig. 7b)**. E-Cad is predominantly localized to the plasma membrane in epithelial cells, whereas in mesenchymal cells it was mainly expressed in the perinuclear region [24, 25]. In the non-treatment group, we observed that E-cadherin was mainly expressed on the membrane. On the contrary, gemcitabine treatment altered translocation of E-cadherin from cellular junction to cytosol. However, pre-treatment with AuNPs prevented to some extent gemcitabine induced translocation of E-cadherin **(Fig. 7b)**. Taken together, these results, demonstrate that AuNPs prevent gemcitabine induced EMT in pancreatic cancer cells by down-regulating expressions of mesenchymal markers and up-regulating the expression of epithelial marker.

### AuNPs suppress cancer stem cell properties

**Figure 8:**
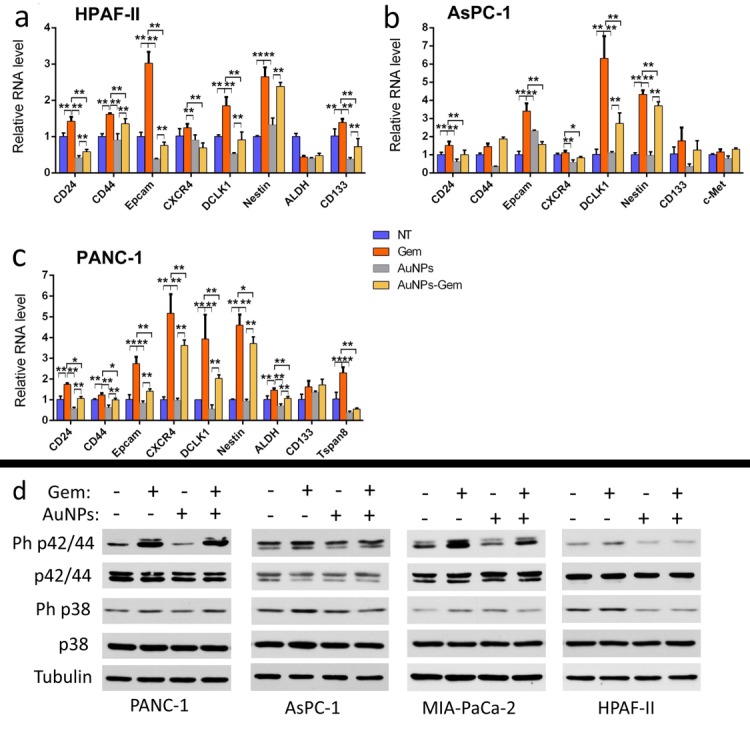
FIGURE 8: AuNPs inhibit the expression of cancer stem cell markers and MAP kinase signaling induced by gemcitabine. PANC-1 **(a)**, AsPC-1 **(b)**, and HPAF-II **(c)** were first starved for 24 h and then divided into four groups for the treatment under the starvation condition: 1) NT group, 2) treated with gemcitabine (100 nM) for 72 h, 3) treated with AuNPs (25 μg/ml) for 24 h, 4) first treated with AuNPs (24 h) and then with gemcitabine (100 nM, 72h). Following the treatment either the total RNA of the cells was extracted and stem cell markers were quantified by qPCR **(a-c)** or the cell lysates were analyzed via western blot **(d)**. All the assays were repeated three independent times. Values are means ±SD and statistical analysis were performed using one-way ANOVA. *P≤0.05, **P≤0.01.

Since EMT was identified as a common regulator of the cancer stem cell (CSC) phenotype having close relationship with drug-resistance in various carcinoma types [7, 26], we investigated whether AuNPs decreased CSC markers and thus reduced cancer cell stemness as a mechanism for gemcitabine sensitization. After treatment with gemcitabine (72 h), AuNPs (96 h), or with the combination therapy first with AuNPs (24 h) and then with gemcitabine (72 h), the total RNA was extracted, transcribed into cDNA, and quantified with qPCR. CD24, CD44 and Epcam are the three main stem cell markers in pancreatic CSCs [27]. Gemcitabine treatment upregulated levels of these markers in three pancreatic cancer cell lines **(Fig. 8a-c)**. However, pre-treatment with AuNPs significantly reduced gemcitabine induced upregulation of these markers. Additionally, we found that pre-treatment with AuNPs could also reverse the gemcitabine-induced upregulation of other stem cell markers [28, 29], such as CXCR4, DCLK1, Nestin, CD133, c-Met, ALDH and Tspan8. These results show that AuNPs can inhibit the up-regulation of stem cell markers induced by gemcitabine, indicating that AuNPs may sensitize the gemcitabine-resistant pancreatic cancer cells to gemcitabine through reversal of the EMT effect and inhibiting the stemness induced by gemcitabine.

### AuNPs reverse the MAP kinase activation

MAPKs are key mediators of cancer cell proliferation and related to drug resistance [30, 31]. In the current study, we were curious as to whether reduction of the MAPK activation could be one of the reasons for the sensitization effect of AuNPs in the pancreatic cancer cell lines. After the treatment with gemcitabine (72 h), AuNPs (96 h), or first with AuNPs (24 h) and then with gemcitabine (72 h), the cell lysates were analyzed by western blot. Treatment with gemcitabine showed significant up-regulation of phosphorylated p42/44 and p38, compared to the NT group. In the AuNPs only treatment group, the MAPK activation was slightly decreased. However, the expression levels of phosphorylated p42/44 and p38 were significantly lowered compared with gemcitabine treatment group **(Fig. 8d)**. This suggests that MAPK activation induced by gemcitabine was downregulated by AuNPs.

## DISCUSSION

Gemcitabine emerged as the standard treatment for advanced pancreatic cancer in the late 1990s. However, due to chemoresistance, no drastic improvement of the medium survival rate was noticed [32, 33]. Moreover, chemotherapy is usually associated with toxicities and adversely effects the quality of life [34]. As such the main aims of the current study were to investigate whether AuNPs act synergetic with gemcitabine in overcoming the chemo-resistance and the possible mechanism behind this.

It has been reported that gemcitabine-resistant cancer cells are more tumorigenic *in vitro* and *in vivo*, and have greater sphere-forming activity than Gemcitabine-sensitive cancer cells [35]. As we can see from our data, AuNPs significantly altered proliferation in PANC-1 and Mia-PaCa-2 in 2D colony **(Figure 2)** and 3D sphere **(Figure 3)** formation assays, which indicates that AuNPs inhibit the tumorigenicity of the pancreatic cancer cells *in vitro*. AsPC-1 originated from pancreas ascites from an adenocarcinoma patient with moderate to high differentiation, while the other two cell lines (PANC-1 and Mia-PaCa-2) were from primary adenocarcinoma tumor with poor differentiation. AsPC-1 has higher levels of N-acetyl glycoproteins and/or glycolipids compared to other cell lines. Since glycolipids and glycoproteins are mainly found in the outer layer of the cellular membrane, it suggested the alteration of membrane compositions in AsPC-1 cells [36]. In addition, compared with PANC-1 and Mia-PaCa-2, AsPC-1 expressed lower levels of pro-angiogenic factors such as COX-2 and VEGF [37]. In our previous study, AuNPs exhibit inhibition effect to pancreatic cells by decreasing the expression of angiogenetic factors including VEGF, FGF and TGF [16]. These might be the reasons that AuNPs did not show significant inhibition ef-fect on AsPC-1 in 2D colony assay compared to on PANC-1 and Mia-PaCa-2 cells. But further research needs to be done and more mechanisms behind the therapeutic effect of AuNPs are required to be verified. Furthermore, AuNPs greatly lower the colony numbers in the AuNPs-gemcitabine combined treatment group compared to the gemcitabine only treatment group **(Figure 6)**. These results suggest that AuNPs possess the potential to overcome therapy resistance by decreasing colony forming ability.

EMT is a process of trans-differentiation of epithelial cells to motile mesenchymal cells. Although it is an integral part of development, wound healing and stem cell behavior, it also contributes pathologically to fibrosis and cancer progression [38]. In the process the cell acquires enhanced migratory capacity, invasiveness, elevated resistance to apoptosis, and enhanced production of ECM components. Finally, the underlying basement membrane is degraded and the resultant mesenchymal cell has the ability to migrate away from the epithelial layer from which it originated [39]. EMT has also been implicated in acquired drug resistance in cancer cells [40]. Thus, targeting EMT and trying to reverse the process might be a potential therapeutic approach to sensitize drug-resistant cells to chemotherapeutics and to inhibit metastasis. Here we investigated whether AuNPs could blunt EMT effect induced by gemcitabine in gemcitabine-resistance pancreatic cancer cells. Western blotting analysis and fluorescence assays revealed that pre-treatment with AuNPs blunts the EMT process induced by gemcitabine and reverses the translocation of E-Cadherin from the membrane to the cytosol back to the membrane. These results suggest that AuNPs sensitize pancreatic cancer cells to gemcitabine by protecting it from gemcitabine induced EMT.

Stemness is believed to be a key factor induced by chemoresistance and tumor recurrence [13, 35]. EMT induced resistance to chemo-therapy is linked to the generation of cancer stem cells, and chemoresistant pancreatic cancer cells express up-regulated stem cell markers consistent with EMT [41]. We hypothesized that the stemness induced by gemcitabine could be inhibited by AuNPs. In our present study we found that when pancreatic cancer cells were treated with gemcitabine, there was an upregulation of several stem cell markers. This is consistent with a published report [29]. However, when the same cells were pretreated with AuNPs prior to gemcitabine treatment, the aforesaid upregulation of stem cell markers was significantly inhibited. These results suggest that AuNPs might prevent the cancer cells' epithelial plasticity, thereby sensitizing the cancer cell to gemcitabine, and consequently inhibiting the proliferation and tumorigenesis.

The mitogen-activated protein kinase (MAPK) cascade is a critical pathway for human cancer cell proliferation, dissemination and drug resistance [42]. Activation of the ERK MAPK pathway is involved in pathogenesis, progression, and oncogenic behavior of human colorectal cancer [43]. Moreover, there is increasing evidence that MAPK is related with chemoresistance [44]. In our previous work, we demonstrated that AuNPs abrogate MAPK-signaling in ovarian cancer cells and pancreatic cancer cells by down-regulating MAPK [16, 17]. Here, we tested the potential of AuNPs for reversing the MAPK signaling induced by gemcitabine in several gemcitabine-resistant pancreatic cancer cell lines. We found that ph-p42/44 and ph-p38 were upregulated after the treatment with gemcitabine. However, pre-treatment with AuNPs decreased this phosphorylation, which suggests that AuNPs sensitize pancreatic cells to gemcitabine and alter the drug resistance of the cell line.

In conclusion, nanomedicine promises to overcome persistent drug resistance. In this study, we observed that AuNPs could reverse the more tumorigenic gemcitabine-resistant pancreatic cancer cells into the less tumorigenic epithelial-like phenotype. Treatment with AuNPs prevented the mesenchymal transition induced by gemcitabine, blunted the stemness and inhibited the potential signaling pathway for the metastasis of cancer. PDAC is characterized by abundant tumor stroma with activated cancer-associated fibroblasts (CAFs) that maintain a dense biophysical meshwork around neoplastic ductal cells [45]. In our previous work, AuNPs were found to inhibit the proliferation and migration of CAFs, thereby further preventing the cross-talk between the pancreatic cancer cells and the CAFs [16]. Thus, accumulating evidence in cancer and other diseases strongly suggests AuNP treatment has the potential to overcome therapy resistance. Our future studies will focus on translating our *in vitro* findings to *in vivo* clinically relevant models of pancreatic cancer.

## MATERIALS AND METHODS

### Reagents

Gold (III) chloride (520918,), Sodium Citrate (C8532), gemcitabine (G6423), and Thiazolyl Blue Tetrazolium Bromide (MTT) (M6494) were purchased from Sigma (St. Louis, MO, USA). Matrigel (CACB354234) and transwell (89235-020) were bought from VWR (Radnor, PA, USA). Alexa Fluor 488-conjugated secondary antibody (R37114) and Alexa Fluor 568-Phalloidin (A12380) were purchased from Life technology (Waltham, MA, USA). The RNA extraction kit is from ZYMO research (R1055, Irvine, CA, US). cDNA transcription kit (1708891) and the qPCR kit (1708882, iQ SYBR Green Supermix) were from Bio-Rad (Hercules, CA, USA).

Anti-E-cadherin (610182) and anti-N-cadherin (610921) were from BD Bioscience ( San Jose, CA, USA); anti-Vimentin (5741), anti-p38(8690), anti-phospho p38(9211), anti-p42/44 (9102), and anti-phospho p42/44(4370) were from Cell Signaling (Danvers, Massachusetts, US); anti-GAPDH(G9545, Sigma, St. Louis, MO, USA), anti-Tubulin(ab18207, Abcam, Cambridge, MA), DAPI( H-1500, Vector Laboratories, Burlingame, CA, USA).

### Cell culture

The human cancer cell lines PANC-1(CRL-14690), AsPC-1(CRL-1682), MIA-PaCa-2 (CRL-1420) and HPAF-II (CRL-1997) were purchased from American Type Culture Collection, and grown in DMEM (10-013-CV, Corning, NY)+10% FBS (16000-044, Life technologies, Carlsbad, CA, USA), RPMI1640 (10-040-CV, Corning)+10%FBS, DMEM+10% FBS+2.5% Horse serum (R55075, Life technologies) and DMEM+10% FBS respectively. 1% Penn-Strep (15140-122, Life technologies) was added to the various culture media.

### Preparation of 20 nm AuNPs

Gold nanoparticles (AuNPs) were synthesized according to the previously published procedure [46] with slight modification. In brief, 5 ml of a 20 mM gold stock solution was added to 180 ml of endotoxin free water in a 500 ml conical flask. The solution was then boiled in a heater equipped with magnetic stirrer to which 15 ml of 1 % (mass/volume) of sodium citrate was added and heating continued. Once the color of the solution changed to dark wine red, the conical flask was kept on a magnetic stirrer overnight.

Synthesized AuNPs were physico-chemically characterized by UV-Visible spectroscopy (UV-Vis), dynamic light scattering (DLS), zeta potential measurements and transmission electron microscopy (TEM). The samples for TEM were prepared by drop coating carbon-coated copper grid with AuNPs solution followed by drying in air. Coated grids were then observed under the TEM microscope. The zeta potential of AuNPs was measured in a Malvern Zeta Sizer Nano instrument using a clear zeta disposable capillary (Malvern DTS1061, Westborough, MA, United States). UV-visible spectra of synthesized AuNPs were recorded in SPECTROStarNano (BMG Labtech Inc, Cary, NC, USA). AuNPs were purified by centrifugation at 10,000 rpm for 20 min at 10°C for cell treatment and concentration of gold in the pellet was calculated after recording the UV spectra of the concentrated pellet [16].

### 2D colony formation

Cells (200 cells/well) were seeded onto 6 wells plates in complete growth medium containing 10% FBS. After 24 hours of incubation, AuNPs and/or gemcitabine at different concentrations were added into the wells and incubated for another 7-10 days after which the colonies were washed with PBS (02-0119-0500, VWR, Radnor, PA, USA) and then stained with crystal violet solution (20% alcohol (v/v) + 0.1% crystal violet (w/v) (B21932, Alfa Aesar, Haverhill, MA, USA)). After washing with water, the colonies were dried and counted with the GelCount (Oxford Optronix, Abingdon, UK). Independent experiments were repeated for at least three times and each time at least triplicate wells were used.

### 3D sphere formation

The sphere formation assay was performed in 24 well plates with matrigel in a bilayer fashion. For the lower layer, 125 μl of the matrigel was mixed with an equal volume of the complete medium containing 10% FBS. 250 μl of the mixture was then added to each of the 24 wells. To generate the upper layer, cells were mixed with the same mixture as the lower layer at a concentration of 600 cells/well (PANC-1 and AsPC-1) or 400 cells/well (MIA PaCa-2). The upper layer was then seeded on the top of the lower layer and allowed to solidify for 30 min. After 30 minutes, various concentrations of AuNPs were added in the complete medium and then added on the top of upper layer. The spheres were observed twice every week. 20-40 days later, the images of the spheres were taken with GelCount and the sphere numbers were counted. Independent experiments were repeated for at least three times and each time at least triplicate wells were used.

### Migration assay

Cells were treated by AuNPs (5 μg/ml or 25 μg/ml) in serum-free medium (serum negative) for 48 h, then washed with PBS, trypsinized and seeded in the transwell insert (8 μm) in a 24 well plate at a seeding concentration of 1×10^5^ cells/well under serum-starved condition. Growth medium containing 1% FBS was added to the lower chamber and the cells were allowed to migrate for 16 h. Cells in the transwell insert were then scraped carefully with cotton swab. After washing with PBS for once, cells on the lower side of the insert filter were stained with crystal violet solution (20% alcohol (v/v) + 0.1% crystal violet (w/v)). After washing with water, the photos of the migrated cells were taken with the camera linked with inverted microscope and the number of cells migrated were counted with Image J software. Independent experiments were repeated for at least three times.

### IC_50_ assay

Cells (1×10^4^/well) were seeded into 24 wells plate in growth medium containing 10% FBS. After 24 h, cells were treated with/without AuNPs (25 μg/ml). After another 24 h, all cells except the non-treatment (NT) group were treated with gemcitabine at different concentrations (range from 100 pM to 10 μM) for 72 h. At the end of the treatment, 1/10 volume of MTT (5 mg/ml in PBS) was added into each well and put back in the incubator for 4 h. After incubation with MTT reagents for 4h, the supernatant was removed and 200 μl DMSO (D4540, sigma Aldrich) was added to dissolve formazan crystals formed in cellular mitochondria. The relative absorption of the dissolved formazan was measured with SPECTROStarNano (BMG Labtech Inc, Cary, NC, USA). Independent experiments were repeated for at least three times and each time, at least triplicate wells were used. IC_50_ value was determined by using Graphpad Prism software.

### Western blotting

Cells were first seeded in 100 mm dishes and 24 h later, all the cells were starved for 24 h with serum negative medium. After the starvation, the medium was replaced with fresh starving medium, and then cells were divided into four groups: 1) Control group or NT group; 2) Gemcitabine group: cells were treated with gemcitabine for 72 h; 3) AuNP treatment group: cells were treated with AuNPs for 96 h); 4) Combination group: cells were first treated with AuNPs (24 h) followed by gemcitabine treatment for 72 h. After the treatment, cells were lysed with RIPA buffer in the presence of proteinase inhibitors (78440, Thermo Scientific, Grand Island, NY). After removing the cell debris by centrifugation, supernatants were collected and concentration of protein in the supernatant was determined with BCA assay (Thermo Scientific, Waltham, MA, USA). 20 μg of protein was loaded in each well of a 10% gel and run. Separated proteins were transferred to a PVDF membrane (1620177, Bio-Rad, Hercules, CA, USA). After blocking with 5% bovine serum albumin (BSA, BP1600-100, Fisher Scientific, Waltham, MA, USA) in TBST for 1 h at 37°C, the blots were incubated with the primary antibodies overnight at 4°C and detected by HRP-conjugated secondary antibodies (Western ECL Substrates, 1705061, Bio-Rad). Independent experiments were repeated for at least three times. The concentrations of the antibodies were as follows: 1:1000 for E-Cadherin, N-Cadherin, vimentin, p42/44, p38 and ph-p38; 1:5000 for ph-p42/44; 1:10000 for a-Tubulin. Secondary antibody dilution factors were 1:10000.

### Immunofluorescence

Cells were seeded on coverslips, and treated with AuNPs/gemcitabine with the same protocol as in the western blotting part. After the treatment, cells were washed with phosphate-buffered saline (PBS) once, and then fixed with 4% paraformaldehyde (J19943K2, Thermo Scientific) at room temperature for 15 mins. This was followed by washing with PBS for 3 times. Cells were permeabilized for 15 mins with 0.2% Triton X-100 (Sigma-Aldrich). After washing with PBS, cells were first blocked with 3% BSA in PBS for 30 min at RT, and then incubated with anti-E-Cadherin antibody (1:200) overnight at 4°C. The primary antibody was detected with Alexa Fluor 488-conjugated secondary antibody (R37114, Life Technologies, Waltham, MA, USA). After washing with PBS, F-actin and nuclei were stained with Alexa Fluor 568-Phalloidin and DAPI separately. Independent experiments were repeated for at least three times. The fluorescence was observed with a Zeiss Axiovert 200 m Inverted Fluorescent Microscope (Axio Observer, Zeiss, Oberkochen, Germany) and images taken.

### Total RNA extraction, cDNA synthesis and quantitative Real-Time PCR

Cells were treated with AuNPs/gemcitabine with the same protocol as in the western blotting part. After the treatment, total RNA of each group of cells was isolated following the manufacturers' instructions (ZYMO research (R1055, Irvine, CA, US). Followed by assessing the concentration of the RNA with SPECTROStarNano (BMG Labtech Inc, Cary, NC, USA), the RNA was reverse transcribed into cDNA with the cDNA Synthesis Kit (Bio-Rad). Quantitative real-time PCR was used to amplify and measure the different stem cell marker genes. The relative quantification of target genes was detected in an ABI PRISM 7300HT Sequence Detection System (Applied Biosystems; CFX Connect, Bio-Rad) and was calculated using the comparative cycle threshold (CT) method (2^−ΔΔCT^) with genes normalized to GAPDH. Each experiment was conducted in triplicate and three separate time points were done for each reaction. The sequences of the primers for each stem cell marker are listed in **Table 1**.

**TABLE 1:** Primer sequences used for quantitative RT-PCR.

**Gene name**	**Sequence: 5′ – 3′**
GAPDH	Fwd: CCC TTC ATT GAC CTC AAC TAC A Rev: ATG ACA AGC TTC CCG TTC TC
CD24	Fwd: CTC CTA CCC ACG CAG ATT TAT T Rev: CGC CAT TTG GAT TGG GTT TAG
CD44	Fwd: CAC CCA AAG AAG ACT CCC ATT C Rev: GCA GTA GGC TGA AGC GTT ATA C
Epcam	Fwd: GAG ATA AAG GAG ATG GGT GAG ATG Rev: AAC GAT GGA GTC CAA GTT CTG
CXCR4	Fwd: CCA CCA TCT ACT CCA TCA TCT TC Rev: ACT TGT CCG TCA TGC TTC TC
DCLK1	Fwd: GGT GGA CTT TCC TTC TCC ATA C Rev: TGG GAG GCC ATC ATC ATT AAC
Nestin	Fwd: CAC TCC AGT TTA GAG GCT AAG G Rev: CCC TCT ATG GCT GTT TCT TTC T
ALDH	Fwd: AGC CCA CAG TGT TCT CTA ATG Rev: GCA GAG CAG AGG AGA TTG TTA T
CD133	Fwd: ACT TGG CTCA GAC TGG TAA ATC Rev: ACT CTC TCC AAC AAT CCA TTC C
Tspan8	Fwd: GGA TGC TGC GGT GCT ATA A Rev: ACA GCT CCT AGG ATA CCT GTC
C-Met	Fwd: GGA GCA CTA TGT CCA TGT GAA Rev: CAC CTC ATC ATC AGC GTT ATC T

## Abbreviations:

AuNP: – gold nanoparticle,
CAF: – cancer-associated fibroblast,
CSC: – cancer stem cell,
DLS: – dynamic light scattering,
EMT: – epithelial to mesenchymal transition,
GF: – growth factor,
NT: – not treated,
PDAC: – pancreatic ductal adenocarcinoma,
TEM: – transmission electron microscopy.
